# Bioimpedance Vector Analysis for Heart Failure: Should We Put It on the Agenda?

**DOI:** 10.3389/fcvm.2021.744243

**Published:** 2021-09-28

**Authors:** Bo Liang, Rui Li, Jia-Yue Bai, Ning Gu

**Affiliations:** ^1^Nanjing University of Chinese Medicine, Nanjing, China; ^2^Nanjing Hospital of Chinese Medicine Affiliated to Nanjing University of Chinese Medicine, Nanjing, China

**Keywords:** bioimpedance vector analysis, heart failure, congestion, risk prediction, management

## Abstract

Heart failure is a clinical syndrome, resulting in increased intracardiac pressure and/or decreased cardiac output under rest or stress. In acute decompensated heart failure, volume assessment is essential for clinical diagnosis and management. More and more evidence shows the advantages of bioimpedance vector analysis in this issue. Here, we critically present a brief review of bioimpedance vector analysis in the prediction and management of heart failure to give a reference to clinical physicians and guideline makers.

## Background

Heart failure (HF) is a clinical syndrome characterized by signs and symptoms associated with abnormal cardiac function and/or structure, resulting in increased intracardiac pressure and/or decreased cardiac output under rest or stress (1, 2). In the developed countries, HF prevalence accounts for about 1~2% of the adult population and sharply rises to more than 10% in people aged 70 or older (3–5). One in six people over the age of 65 who have difficulty breathing due to fatigue or exertion and receive primary care have unidentified HF (6, 7). The lifetime risk of HF at age 55 years is 33% for men and 28% for women (8). Fortunately, based on the temporal trend data of inpatients, the incidence rate of HF may be decreasing (9, 10). At present, the definition of HF is limited to the stage of obvious clinical symptoms and signs. Before the clinical symptoms and signs become obvious, patients may show asymptomatic structural or functional cardiac abnormalities, which are precursors of HF (11). The identification of precursors is important since they are associated with poor prognosis (12), and initiation of treatment at the precursor stage can reduce mortality in asymptomatic patients with reduced left ventricular ejection fraction (12, 13).

Congestion in HF is often underdiagnosed. Although international guidelines force physicians to assess the fluid accumulation in patients with HF (1, 11, 14–16), most of them are still congested when they are discharged from intensive care units and/or cardiology (17, 18). When HF patients are discharged from the hospital with congestion, both mortality and readmission rates increase (19). Early detection of fluid retention is challenging in HF. Recently, bioimpedance vector analysis (BIVA) has emerged as a new tool able to evaluate congestion (20). The electrical impedance field is also corroborated by implanted devices able to assess intrathoracic impedance and communicate data directly via telemonitoring. Here, we present a brief review of BIVA in the prediction and management of HF to give a reference to clinical physicians and guideline makers.

## BIVA

**Figure 1:**
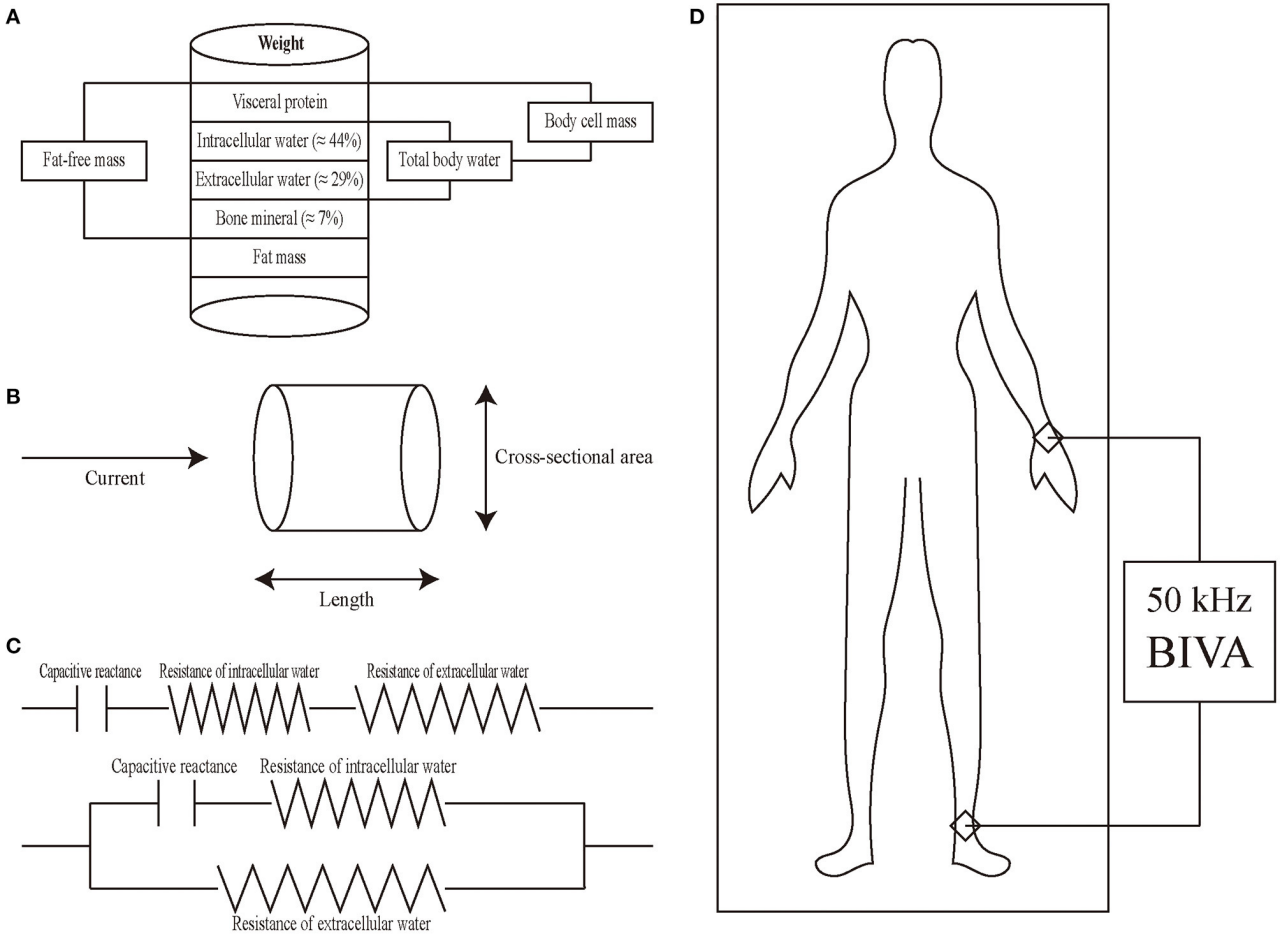
Introduction of BIVA. **(A)** Schematic diagram of intracellular water, extracellular water, total body water, body cell mass, and fat-free mass. **(B)** Principles of BIVA from physical characteristics to body composition. Cylinder model for the relationship between impedance and geometry. The resistance of a length of homogeneous conductive material of uniform cross-sectional area is proportional to its length and inversely proportional to its cross-sectional area. **(C)** The human body consists of resistance and capacitive reactance connected in series (upper) or in parallel (lower). **(D)** Placement of electrodes in BIVA and total body water is measured by the ankle-wrist bioimpedance method.

BIVA is a portable, non-invasive, simple, and easy auxiliary examination, which dose not expose patients to ionizing radiation and is not affected by differences between observers and investigators (21). BIA works well in healthy subjects and subjects with chronic diseases and is suitable for different races, genders, and ages (22, 23). The indicators of the liquid state include extracellular water, intracellular water, and total body water (Figure 1A), which can reflect the distribution of body water composition inside and outside cells. The basic method is to regard the human body as a conducting cylinder. The electrical properties of the fluid inside and outside the human cells and the cell membrane are different so that the impedance generated by the current passing through is different. Then the resistance and capacitive reactance under different currents are measured by the external circuit (Figure 1B). Resistance is mainly determined by the electrical properties of intracellular and extracellular fluids, while capacitive reactance is mainly determined by the capacitive properties between cell membranes (22, 23). At present, there are many kinds of electrical circuits to describe the behavior of biologics, the most common and simplest are series connection and parallel connection (Figure 1C). Generally, both resistance and capacitive reactance can be measured at the same frequency [mostly 50 kHz (24), Figure 1D].

Although BIVA has many advantages, there are aspects that need to be paid attention to in our clinical application. Firstly, BIVA measurement of body composition is easily affected by body fluid changes (such as drinking, diet, diarrhea, and exercise) (32, 33). Secondly, in order to avoid measurement error, it is necessary to professionally calibrate BIVA equipment regularly for accurate measurement. In addition, although 50 kHz is used as the detection frequency in most cases, different frequencies can result in different measurement (34). It is necessary to study the sensitivity and specificity of different frequencies to the human constitution through a large sample study of the population, so as to select the best detection frequency. Finally, BIVA results are also affected by the extremes of body mass index, which is inevitable. It is not recommended to use BIVA for routine evaluation of such patients until accuracy of the BIVA algorithm can be further verified.

## Clinical Application of BIVA

BIVA identifies the components of bioelectrical impedance and interprets them as a function of fluid status (35) (Figure 1D). Serum colloidal osmolality is the main determinant of peripheral hyperemia in patients with HF by BIVA using a single alternate current frequency of 50 kHz (36), and the advantages of BIVA are it is easy, fast, low cost, and non- invasive (25, 37).

A retrospective study of 706 hospitalized patients with acute HF (AHF) showed that the higher the hydration state evaluated by BIVA, the longer the hospital length of stay, suggesting congestion is an independent predictor of the total length of hospital stay in acutely decompensated HF patients (26). BIVA was more accurate than BNP in detecting peripheral congestion in AHF (the area under the curve (AUC) was 0.88 vs. 0.57 respectively; *P* < 0.001) (25). In addition to AHF, BIVA also has good diagnostic efficacy for chronic HF (CHF). BIVA was more accurate than BNP in detecting peripheral congestion in CHF (AUC was 0.89 vs. 0.68, respectively; *P* < 0.001) (25). A prospective trial from Italy indicated that BIVA can effectively predict the total events at admission and discharge (AUC was 0.56 and 0.57, respectively) (27). In addition to diagnosis, BIVA is also relevant to the management of patients with HF (38, 39). In HF patients, especially those with AHF, a physical examination reflects the degree of rales and lower limb edema, thereby lays the foundation of clinical management. Another study involving 51 emergency patients with suspected AHF from Italy confirmed that the AHF group suffers from greater initial fluid status predicted by BIVA compared with the control group (28). In addition, the hydration state measured by BIVA in the AHF group was significantly decreased 72 h after diuretic medication treatment and at discharge (28). Routine laboratory testing, such as brain natriuretic peptide (BNP)/N-terminal pro BNP (NT-pro BNP) (2, 40), is of great clinical significance for the reaction of peripheral fluid accumulation. BIVA significantly improved the prediction ability of cardiovascular events at 3 months (AUC = 0.97) when combined with clinical symptoms and signs (27). In a small preliminary study of 54 ambulatory patients with HF, BIVA also distinguished between stable and unstable HF. Specifically, patients with stable HF have significantly lower impedance measured fluid load ratio (Rz/H) and cardiac stress biomarkers, such as NT-pro BNP, than patients with unstable HF (29). The data from 184 patients with AHF and 252 patients with CHF with a median follow-up of 463 days indicated that the optimum cut-off values for death were estimated plasma volume status >5.3 dL/gr, BNP > 441 pg/mL, hydration index evaluated by BIVA > 73.8%, and blood urea nitrogen/creatinine ratio (BUN/Cr) > 25 (30). The mortality of patients with all four indicators above the optimum cut-off values was 93% higher than that of patients below the optimum cut-off values (30). In addition, an in-hospital resistance variation (dR/H) increase of more than 11 Ω/m was related to overall survival (27).

The number of patients with cardiac implantable electronic devices (CIEDs) is increasing all over the world. The use of BIVA in patients having CIEDs is limited because of concerns about electromagnetic interference. However, a study of 200 patients from France indicated that there were no significant changes in battery lead impedance, voltage, or pacing thresholds during BIVA (31). In addition, no changes in CIEDs were found at 0.5 and 1 year of follow-up (31), suggesting that BIVA performance in patients having CIEDs is secure. A study that included 43 patients from Brazil demonstrated that there were significant reductions in BIVA parameters, including reactance, resistance, and measurements after CIEDs were implanted (41).

In conclusion, current evidence shows that BIVA can be used not only to evaluate the effect of HF treatment but also to predict the total length of hospital stay and the total events of admission and discharge in patients with HF (Table 1). In addition, it is safe and reliable for CIEDs patients (Table 1).

**Table 1:** Summary of main findings.

**First author**	**Country**	**Design**	**Population**	**Main findings**	**References**
Massari	Italy	Retrospective study	487 AHF and 413 CHF	BIVA is an easy, fast technique to assess peripheral congestion, and is even more accurate than BNP in HF patients.	(25)
Massari	Italy	Retrospective study	706 AHF	The higher the hydration status, the longer the hospital length of stay.	(26)
Santarelli	Italy	Prospective, multicenter, observational study	336 AHF	An increase of resistance variation >11 Ω/m during hospitalization was associated with survival. When combined with clinical signs, BIVA showed a very good predictive value for cardiovascular events at 90 days (AUC 0.97).	(27)
Somma	Italy	Not reported	51 ADH	The initial fluid status predicted by BIVA was greater in the AHF group than the controls.	(28)
Gastelurrutia	Spain	Not reported	54 HF	There were statistical differences between the stable and non-stable HF patients in the ratio of impedance-measured fluid overload.	(29)
Massari	Italy	Retrospective study	184 AHF and 252 CHF	The optimal cut-off for death occurrence were hydration index evaluated by BIVA > 73.8%.	(30)
Chabin	France	Prospective study	200 CHF	BIVA has no interference in patients equipped with CIEDs.	(31)

## Future Perspectives

Patients with HF are usually admitted to hospital because of symptoms and/or signs of congestion, and fluid overload is the most common cause of readmission (42). In the clinical management of HF patients, adequate fluid volume could improve the short- and long-term outcomes, but most physicians' efforts are concentrated on the identification of peripheral edema, signs of lung congestion, and ascites. Indeed, these are late signs of congestion as they indicate advanced accumulation of fluids. The prognosis of patients at this stage is not ideal. Clinicians should set biomarker and indicator thresholds in order to obtain a sufficient fluid state (38). Rapid and reliable hydration detection provides the possibility of targeted therapy for AHF patients, thus cutting down the length of total hospital stay and treatment costs (26). The simple BIVA mode allows vector displacement as therapeutic feedback to detect, monitor, and even control congestion status (43). BIVA may be used as a routine bedside body fluid assessment and management method (44), however, BIVA is not recommended in the current HF guidelines.

BIVA can classify and rank hydration status without the influence of body weight through comparing with the healthy people norms. These different classifications (normal, under-, and over-) and rankings (change relative to pre-treatment) have a wide range of applications in assessing and managing the progress and prognosis of over-hydration (45). The increase of BNP in HF patients from discharge to 1-month follow-up is a helpful prognostic signature for predicting readmission (46). As complementary to BNP, BIVA can provide more accurate prognosis information for patients with HF (30). This could help clinicians to better manage these patients and further reduce the subsequent cardiovascular events (47).

Limitations of BIVA include that it cannot report the bodyweight that would indicate normal fluid status (23). As it requires the application of adhesive electrodes, sweat, hairiness or a patient's inability to cooperate might affect the correct placement of electrodes, thus preventing BIVA measurement. Thirdly, whole-body measurement can provide data indicating excess volume, but its location is not clear. Lung echocardiography can solve this problem jointly (48, 49), but it depends on the physicians to determine the location of the abnormal fluid accumulation (39). Finally, combined with clinical signatures, such as BNP/NT-pro BNP (50) and liver stiffness (51), BIVA may improve the ability to the diagnosis and evaluation of HF, especially AHF.

## Conclusions

For patients with HF, especially AHF, accurate volume assessment is necessary for appropriate management. Although symptoms and signs are the first to be evaluated, the information they provide is still limited. BIVA may make up for this deficiency, though it is not perfect. This challenge can be addressed when physicians integrate clinical and auxiliary assessment.

## Author Contributions

BL and NG conceived, designed, or planned the idea. BL drafted the manuscript. NG revised the manuscript. All authors collected and read the literature, read, and approved the final manuscript.

## Funding

This work was supported in part by Research and Practice Innovation Plan for Postgraduates of Jiangsu, China [KYCX21_1641], National Natural Science Foundation of China [81774229], Jiangsu Universities Nursing Advantage Discipline Project [2019YSHL095], and Jiangsu Leading Talent Project of Traditional Chinese Medicine [Jiangsu TCM 2018 No. 4].

## Conflict of Interest

The authors declare that the research was conducted in the absence of any commercial or financial relationships that could be construed as a potential conflict of interest.

## Publisher's Note

All claims expressed in this article are solely those of the authors and do not necessarily represent those of their affiliated organizations, or those of the publisher, the editors and the reviewers. Any product that may be evaluated in this article, or claim that may be made by its manufacturer, is not guaranteed or endorsed by the publisher.
